# A Biomimetic Silk Fibroin/Sodium Alginate Composite Scaffold for Soft Tissue Engineering

**DOI:** 10.1038/srep39477

**Published:** 2016-12-20

**Authors:** Yiyu Wang, Xinyu Wang, Jian Shi, Rong Zhu, Junhua Zhang, Zongrui Zhang, Daiwei Ma, Yuanjing Hou, Fei Lin, Jing Yang, Mamoru Mizuno

**Affiliations:** 1 State Key Laboratory of Advanced Technology for Materials Synthesis and Processing, Wuhan University of Technology, Wuhan 430070, People’s Republic of China; 2 Biomedical Materials and Engineering Research Center of Hubei Province, Wuhan University of Technology, Wuhan 430070, People’s Republic of China; 3 Hubei Key Laboratory of Quality Control of Characteristic Fruits and Vegetables, Hubei Engineering University, Xiaogan 432000, People’s Republic of China; 4 Department of Machine Intelligence and Systems Engineering, Faculty of Systems Science and Technology, Akita Prefectural University, Akita 015-0055, Japan; 5 Life Science Technology School, Hubei Engineering University, Xiaogan 432000, People’s Republic of China; 6 School of Foreign Languages, Wuhan University of Technology, Wuhan 430070, People’s Republic of China

## Abstract

A cytocompatible porous scaffold mimicking the properties of extracellular matrices (ECMs) has great potential in promoting cellular attachment and proliferation for tissue regeneration. A biomimetic scaffold was prepared using silk fibroin (SF)/sodium alginate (SA) in which regular and uniform pore morphology can be formed through a facile freeze-dried method. The scanning electron microscopy (SEM) studies showed the presence of interconnected pores, mostly spread over the entire scaffold with pore diameter around 54~532 μm and porosity 66~94%. With significantly better water stability and high swelling ratios, the blend scaffolds crosslinked by 1-ethyl-3-(3-dimethylaminopropyl) carbodiimide (EDC) provided sufficient time for the formation of neo-tissue and ECMs during tissue regeneration. Fourier transform infrared spectroscopy (FT-IR), X-ray diffraction (XRD) results confirmed random coil structure and silk I conformation were maintained in the blend scaffolds. What’s more, FI-TR spectra demonstrated crosslinking reactions occurred actually among EDC, SF and SA macromolecules, which kept integrity of the scaffolds under physiological environment. The suitable pore structure and improved equilibrium swelling capacity of this scaffold could imitate biochemical cues of natural skin ECMs for guiding spatial organization and proliferation of cells *in vitro*, indicating its potential candidate material for soft tissue engineering.

Tissue engineering, which aim to reconstruct living tissues for repairing or replacement of damaged or lost tissues/organs of living organisms, is an increasing concern in the life sciences^1^. Design of artificial ECMs is very important in tissue engineering, because suitable ECMs play a pivotal role in supporting cell survival, migration, and differentiation. Also, ECMs have multiple functions, such as serving as an adhesive substrate, provision of structure, presentation and storage of growth factors, and detection of signals^2^. Current approaches are based upon mimicking the complex ECMs consisting of a network of proteins, such as collagens, elastin, proteoglycans, glycoproteins, and glycosaminoglycans^3^. The three-dimensional biodegradable scaffolds provide physical support as a necessary template or matrix for cell attachment and differentiation^4^. They also guided the necessary proliferation of cells into the targeted functional tissue or organ. Therefore, scaffolds using protein and polysaccharides mimicking the ECMs significantly enhance cell attachment, proliferation, and differentiation for tissue regeneration.

Silk from silkworms, *Bombyx mori* cocoons, is mainly composed of sericin and fibroin. Its unique properties which include reported low immunogenicity, impressive mechanical properties, biocompatibility, easy fabrication, a wide range of degradation rates have made silk fibroin became popular biomaterials^5^,^6^,^7^. SF scaffolds have been investigated recently to explore their potentials in soft tissue engineering applications such as repairing ligaments^8^, cartilage^9^, primary nerve^10^ and skins^11^. Alginates are linear copolymers composed of two uronic acids, β (1-4) linked D-mannuronic acid (M) and α (1-4) linked L-guluronic acid (G). They are extracted from native brown seaweed (Phaeophyceae) and provide strength and flexibility to the algal tissues^12^. SA has been investigated for biomedical applications such as drug delivery^13^, living cell and bioactive protein encapsulating matrices^14^, wound dressing^15^, bone regeneration^16^, cartilage repairing^17^ and 3D bioprinting^18^. Especially, a report have suggested that certain alginate dressings can enhanced wound healing by stimulating monocytes to produce elevated levels of tumor necrosis factor-α (TNF-α)^19^. This simulation is advantageous to repairing damaged soft tissue such as impaired skin.

SF is brittle on its own and SA has high swelling ability which causes it to get easily deformed. In order to improve performance of such natural polymers and expand the applied range, they has been blended with various of other polymers which include both natural macromolecules like collagen, gelatin, elastin, chitosan, hyaluronic and sodium carboxymethyl cellulose as well as synthetic molecules like polyacrylamide, polystyrene, polyurethane, poly (L-lactide), polyvinylalcohol, poly ethylene glycol^11^,^20^,^21^,^22^,^23^,^24^,^25^,^26^,^27^,^28^,^29^,^30^,^31^. Those blend scaffolds with more superior properties were employed for various tissue engineering applications. Meanwhile, natural polymers have shown superiority in biomedical applications since they have proven to be most compatible with the native ECMs. SF and chitosan polyelectrolyte complex porous scaffolds not only showed a higher compressive strength and modulus, but also supported the growth and adhesion of feline fibroblasts^11^. The SF/keratin based blended scaffolds enhanced significantly L929 fibroblasts cell adhesion and cell proliferation^26^. The chitin/chitosan, fucoidan and alginate blended hydrogel showed clearly that it was able to absorb fetal bovine serum (FBS) and fibroblast growth factor (FGF-2), which stimulate proliferation of human dermal fibroblast cells (DFCs) and dermal micro-vascular endothelial cells (DMVECs)^28^.

Despite the compatibility, all polymers were insufficient in delivering the desired performances in one or more aspects. Herein, SF and SA were chosen to prepare novel composite scaffolds because in theory the blends would enhance the shortcomings of pure silk scaffolds, and made its potential for damaged soft tissue reconstruction. A suitable scaffold should not merely imitate the composition of the native tissue but mimicking or enhancing the performance and structural performance is necessary. So far, there have been studies on beneficial properties of SF/SA blended films and hydrogels. There was a study reporting SF/SA blend films with globular micro-structure exhibited suitable water vapor transmission, swelling capacity, mechanical properties and non-cytotoxity for wound dressings^32^. In other case studies, the SF/SA hydrogel scaffolds crosslinked by Ca^2+^ were used for recreating artificial stem cell niche^33^, and another novel SF/SA hybrid scaffolds offered new and important options to the needs related to biomineralization in tissue engineering^34^.

Nevertheless, the SF/SA blended materials above weren’t studied for meeting the ability to repair damaged soft tissue, a desirable scaffold with superior performance should be further studied in more details. Gaining the ability to control the water stability and microstructure in the scaffolds is necessary to develop the novel 3D scaffolds. Furthermore, cytotoxicity, cell adhesion, viability, proliferation and biocompatibility *in vitro* in this novel SF/SA scaffold has not been extensively studied, thus, such a biomimetic scaffold need to be further investigated.

In light of the above circumstances, the biomimetic composite SF/SA scaffolds with long-term stability and regular pore structure were constructed using EDC as a crosslinking agent by a freeze-dried method. The mechanism of interactions among SF, SA and EDC was elucidated to explain the enhanced stability in the crosslinked blend scaffolds. For the purpose of investigating the effect of preparation conditions on microstructure of scaffolds, the morphology and microstructure of the scaffolds fabricated in different conditions were observed by SEM. Furthermore, the stability in phosphate buffered saline (PBS), swelling behaviors and structure of scaffolds were fully discussed to evaluate the characteristic. Moreover, the cytotoxicity and cell behaviors in a series of scaffolds were investigated in detail.

## Results and Discussion

### Preparation of the SF/SA scaffolds and mechanism of crosslinking reaction

**Figure 1:**
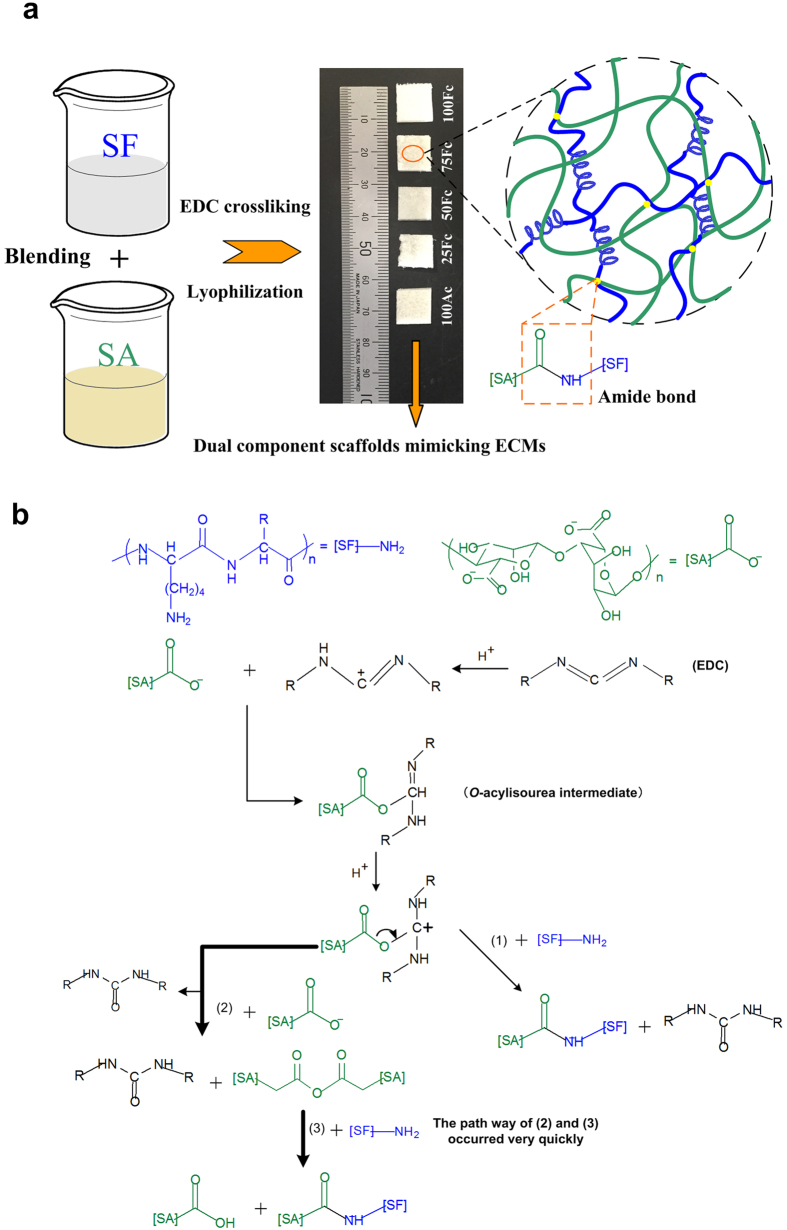
Procedure schematic and EDC reaction mechanism. (**a**) Schematic describling a strategy of preparation the SF/SA scaffolds. (**b**) EDC reaction mechanism schematic with SA and SF: (1) reaction with amino groups, (2) reaction with a nearby ionized carboxyl group and (3) quickly form amide bond when amine is present.

The blend scaffolds were fabricated from aqueous solution of 2 wt% SF and SA solutions at weight ratios of 100/0, 75/25, 50/50, 25/75 and 0/100 (see “Methods” for details; Fig. 1(a)). In order to stabilize the scaffolds against water or solvent, EDC was used as a crosslink agent in this study. EDC is a water soluble reagent for crosslinking two proteins or for activation of carboxylic acids for reaction with ligands containing amino groups. It is widely and effectively used in protein chemistry because resulting linkage contains only an amide bond conjunction, no residues remain in the crosslinked protein^35^. The resulting composite scaffolds were termed accordingly, 100Fc, 75 Fc, 50 Fc, 25 Fc, 100Ac (Fig. 1(a)). These scaffolds seemed to be white sponges, which can be resistant to the water. According to the previous reports^35^,^36^, reaction mechanism among EDC, SF and SA was hypothesized as shown in Fig. 1(b). Firstly, the O-acylisourea intermediate were formed through reaction between ionized carboxyl groups and carbocation in the EDC molecule. And then this activated carboxyl group will change into a carbocation, which followed by attack of various bases in the mixture system. The first possible reaction (i) will be with an amino group in SF molecular, which produces a urea byproduct and an amide bond between SF and SA (step (1) of Fig. 1(b)). The second possible reaction (ii) since an ionized carboxylate group is a very strong base, its reaction with carbocation will form an anhydride followed by a rapid reaction with an amino group to form an amide bond and a urea byproduct (steps (2) and (3) of Fig. 1(b)). Here, N-hydroxysuccinimide (NHS) may also be added to create a more stable amine-reactive NHS ester intermediate, which improves reaction efficiency^37^, and the MES creates a faintly acid condition to be beneficial for the crosslink reaction. The crosslinking reaction occurs facilely around pH 5. Finally, a three dimensional scaffolds with SF chains interpenetrated in SA chains was obtained. The extension of molecular chain and the increase of the crosslinking density in porous scaffolds could lead to stability at physiological environments.

### Microstructural analysis

**Figure 2:**
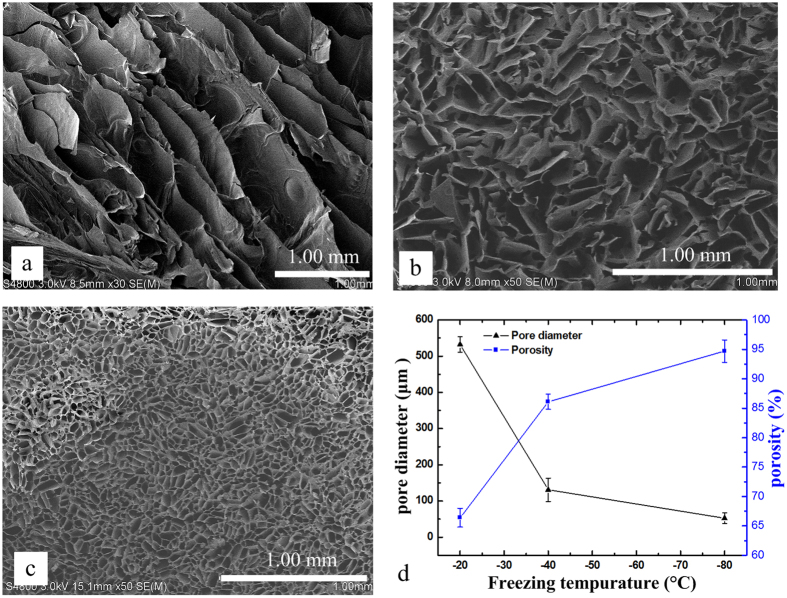
SEM images of the 50Fc samples with different freezing temperature (**a**) −20 °C, (**b**) −40 °C, (**c**) −80 °C, and (**d**) pore diameter and porosity of samples.

**Figure 3:**
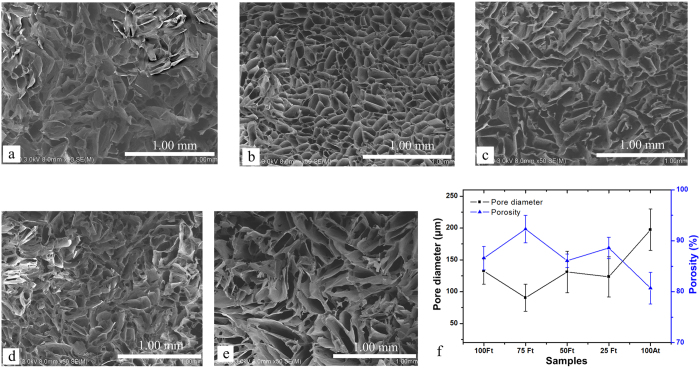
SEM images of the different blend ratios samples with freezing temperature −40 °C (**a**) 100Fc, (**b**) 75Fc, (c) 50Fc, (**d**) 25Fc, (**e**) 100Ac, and (**f**) pore diameter and porosity of samples.

The microstructure characteristic of the blend scaffolds samples which were produced in different freezing temperatures was studied by SEM (Figs 2 and 3). The images of scaffolds demonstrated a continuous phase and interconnected network porous structure in all the blend scaffolds, with mean pore diameters in the range 54~532 μm and porosity in the range 66~94% through changing the blend ratio and freezing temperature. As shown in Fig. 2b, the 50Fc scaffolds showed thin-layer structure with many long and narrow pores inside under −20 °C freezing environment, the pore diameter became smaller with reduction of freezing temperature. With the lower freezing temperature, the pore diameter of 50 Fc decreased dramatically from 532 to 54 μm, which attributed to the size of ice crystals acted as porogens in the freezing process^38^. At the same time, the scaffolds showed more regular and uniform pore morphology under lower temperature. In the freezing process of water-soluble polymer system such as SF solutions, the size of ice crystals is related to the freezing temperature and freezing rate closely, hence freezing process dictated the features of the pores once the scaffold was lyophilized^39^.

By introducing SA into the scaffolds, the pore diameter of blend scaffolds appeared to be smaller than the pure ones. What’s more, addition of the SA created more subordinate pores in the wall of the main pores, which can be seen in the Fig. 3b~d. The mean pore diameter of 75Fc (−40 °C) scaffold was 91 μm, which was the smallest among all the samples under −40 °C. The porosity percentage of 100Fc, 75Fc, 50Fc, 25Fc and 100Ac samples were 85%, 92%, 86%, 88% and 81%, respectively. These results demonstrated the porosity of the 75Fc and 25Fc blend scaffolds was higher than pure scaffolds. This fact could be related to the formation of numerous small pores after adding the SA which could enhance porosity. The results of B.B Mandal *et al*.^25^ were in agreement with this aspect. Consequently, the pore size and porosity of scaffolds was dependent on the ratio of the SA added and freezing temperature, suitable pore structure which is appropriate for fibroblasts growth and proliferation can be obtained by adjusting blend ratios and freezing temperature.

### Stability and swelling behavior

**Figure 4:**
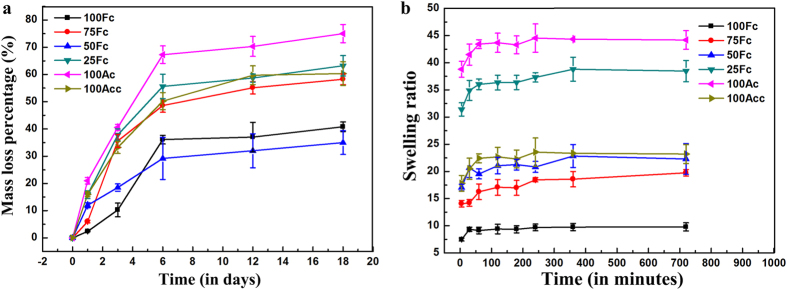
(**a**) Mass loss of various blend ratio scaffolds, (**b**) Swelling ratio (w/w) of samples in water at 37 °C against time.

In order to achieve feasibility of application in tissue engineering, these scaffolds should be stable and should not leach out under physiological conditions. Thus, the weight loss of the scaffolds in the PBS at 37 °C was carried out to evaluate the stability of the blend matrices. Figure 4(a) showed the mass loss of various blend ratios EDC crosslinked scaffolds after 18 days. After EDC crosslinking, the mass loss of all the samples dropped dramatically from ~90% (the uncrosslinked scaffolds’ data were shown in the Fig. S2) to ~10% after 24 h, the initial rate of mass loss of the 75 Fc, 50 Fc and 100Fc was much lower than the other crosslinked ones. The results also showed that the final mass loss ordered as 100Ac > 25Fc > 100Acc > 75Fc > 100Fc > 50Fc, the minimum mass loss was 35.0 ± 4.4%, which was observed in the 50Fc sample. Because EDC crosslink had little effect on enhancing the stability of SA scaffolds, here Ca^2+^ crosslinking method was adopted to improve the stability of SA scaffolds for further investigation. With ongoing immersion time, all the samples kept the integrity of apparent morphology except the 100Ac. This result suggested that the SF/SA blend ratio of 50/50 was desirable to form stable scaffolds with EDC crosslinking. Higher weight loss rate in 100Ac after crosslinking may be ascribed to lack of active groups reacted with the EDC.

The swelling ability of scaffolds plays important role in repairing injured tissues and tissue regeneration. A high swelling ratio is beneficial to transit the nutrition and wastes. Figure 4(b) showed the swelling ratio of the different ratios scaffolds at 37 °C in determined intervals. The scaffolds swelled rapidly in water and attained equilibrium within 6 h. Water uptake by scaffolds increased with time until they obtained equilibrium. The swelling ratio was found to be related to the composition in the scaffolds. It was observed that with augment of SA content, the swelling ratio increased significantly because SA swells more than crosslinked SF. The blend scaffolds of 25Fc achieved a maximum swelling ratio of 38.81 due to the superior water retention capacity of polysaccharides. However, the pure SA with highest swelling ratio of 44.19 because of its larger diameter pores, excellent hydrophilic and unstability in the water. Ca^2+^ crosslinking reduced the swelling ratio of 100Ac. Meanwhile, the blend scaffolds of 75Fc sample showed lowest swelling ratio of 19.70 in the blend scaffolds, it was can be explained that the more intense crosslinking impeded mobility of the polymer chains, which in turn hindered the movement of water, what’s more, there were less SA content in the 75Fc sample. In the present study it suggested that EDC crosslinking reaction between SF and SA decreased the weight loss effectively to stabilize the SF/SA blend matrix against water. At the same time, it was clear that the suitable swelling ratio was obtained through adding different SA contents.

### Conformational structures analysis

**Figure 5:**
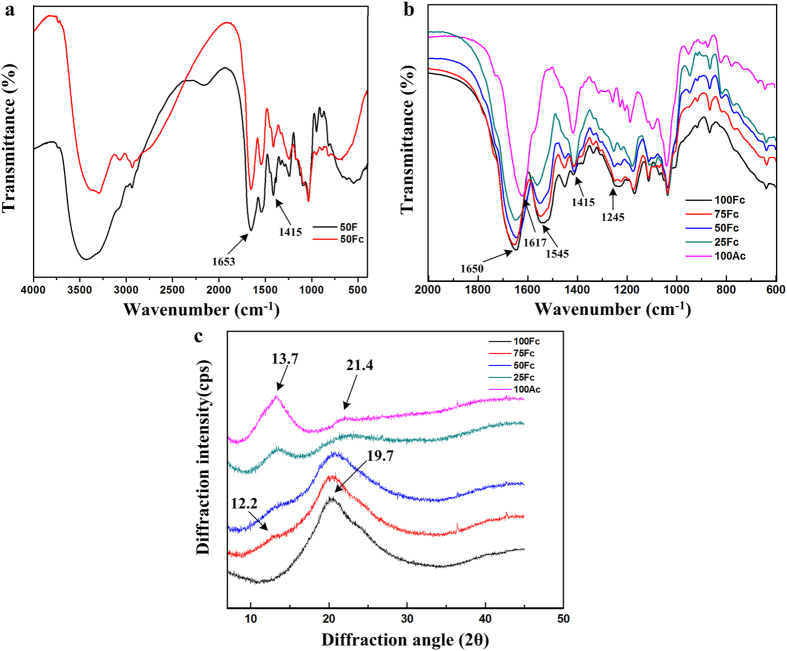
(**a**) FTIR spectroscopy of 50 F and 50Fc scaffolds, (**b**) FTIR spectroscopy of various blend ratio scaffolds crosslinked by EDC, (**c**) XRD patterns of various blend ratio scaffolds.

FTIR spectroscopy has been a useful tool to investigate positions of the amide bonds which are sensitive to the molecular conformation of SF. FTIR spectra of uncrosslinked blend scaffold 50 F (black line) and EDC crosslinked 50Fc (red line) were shown in Fig. 5(a), the FTIR spectra of blend scaffolds with different composition crosslinked with EDC in the range of 600–2000 cm^−1^ were shown in Fig. 5(b). From the spectra, the positions of peaks before and after 50Fc crosslinked by EDC were located at the similar points, but the area of –OH bands (3700–3100 cm^−1^) is much smaller for crosslinked sample due to the loss of bonded water after crosslinking. What’s more, the characteristic peak of carboxyl around 1415 cm^−1^ became smaller than the uncrosslinked one, because EDC crosslinking reaction consumed carboxylic group from the polymers.

These results demonstrated the SF/SA mixture could reacted with EDC in the mild condition. Sinokowska *et al*.^40^ reported that the peak position of FTIR spectra for collagen crosslinked with EDC were the same as those of uncrosslinked collagen, because the secondary structure of collagen was not destroyed. Results of present study were consistent with the previous ones.

The FTIR spectral region between 1700 to 1500 cm^−1^ is assigned to the fibroin peptide backbone of amide I (1700–1600 cm^−1^) and amide II (1600–1500 cm^−1^) absorptions, and the amide III region was from 1350 to 1200 cm^−1^^41^. The pure fibroin scaffolds showed the characteristic peaks of silk I at 1650 cm^−1^, 1540 cm^−1^ and 1243 cm^−1^ (Fig. 5(b), black line). The structure of pure SA was also studied from the FTIR spectral. Generally, the peaks around 1610 cm^−1^, and 1408 cm^−1^ were attributed to the asymmetric and symmetric stretching of carboxylate –COO^–^ respectively, and the peaks around 1034 cm^−1^ belonged to the O–H bending, which was in accordance with the literature for SA^42^. The blending scaffolds presented a spectrum similar to the 100Fc spectrum, but with the presence of SA absorption bands, slightly shifted to lower wavelengths at amide I with increase of SA content compared to pure SF spectrum. The bands related to amide I and amide III of the blend scaffolds overlap with SA characteristic groups, which can induce errors when analyzing the spectra at these specific wavelengths. However, the amide II bands were clearly visualized in the blend spectra, the peak at 1545 cm^−1^ in all the blend curves were signed to the silk I conformation. Accordingly, introduce of EDC could lead to formation of chemical bands between SF and SA macromolecules, but didn’t change the conformation structure of SF in the blend scaffolds.

Conformational changes in different blend ratio scaffolds were also determined by XRD analysis (Fig. 5(c)). A total of 100Fc scaffold showed an arc-shaped diffraction peak at approximately 19.7°, representative of silk I structure in the scaffolds^9^. SA typical halos were observed at 13.7° and 21.4° (magenta line), corresponding to 6.45 and 4.42 Å, respectively^43^. All the blend scaffolds exhibited obvious diffraction peaks around 13.4° and 20°, which demonstrated SA components distinctly existed in the SF/SA blending scaffolds, and the crystalline structure of SF was also mainly the silk I structure based on the previous studies of researchers, the red line showed weak diffraction at around 12.2°, which also assigned to silk I. All the peaks were broad and had low intensity, which were indications of low crystallinity of the prepared scaffolds. With increasing of SA content in the blend scaffolds, the intensity of SF characteristic peak around 20° was becoming weaker and weaker, while the SA characteristic peak around 13.7° appeared clearly in the 25Fc scaffold. These changes were ascribed to increasing interaction between SF and SA molecules. A higher number of SA molecules and amide bond formed due to EDC crosslinking reaction hindered self-assembly of SF, leading to crystalline structure of the blend scaffolds decreased. Hence, XRD results confirmed co-existence of two substances in the blend scaffolds, the addition of SA in the EDC crosslinked composite scaffolds didn’t have influence on the SF structure.

### Cytotoxicity of scaffolds

**Figure 6:**
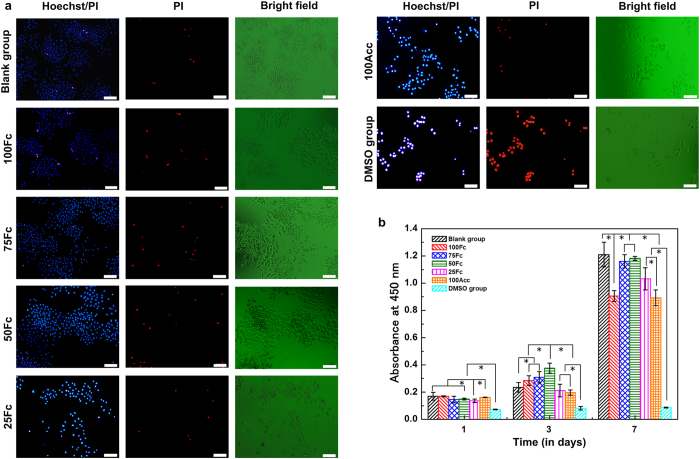
Morphology and proliferation of L929 cells in the scaffolds extract media. (**a**) The cells growing in the extracted media of different samples after 4-day culture were 33342 and PI fluorescent dye, Scale bars, 100 μm. (**b**) the cell viability assessed by CCK-8 assay after 1, 3, 7-day exposure with scaffolds extract media. * denotes statistically significant differences. (n = 6 per group, p < 0.05).

At first, the cytotoxic activity of the scaffolds was evaluated using extraction method. Figure 6(a) showed photomicrographs of L929 cells stained with Hoechst 33342 and PI fluorescent dye after exposure of the cells to extracted media after 4-day culture. Hoechst 33342 and PI staining was used to confirm apoptosis cells and dead cells^44^. In this case, the apoptotic cells, characterized by condensed nuclei or fragmented nuclei, and the dead cells were assigned to the red colour, were observed through fluorescence microscope. Numbers of apoptotic cells and dead cells increased along with the increase of SA content. And there were more normal cells in the 100Fc, 75Fc and 50Fc sample extracted media. Compared to the blank group, the 100Fc, 75Fc and 50Fcsample exhibited no significant differences. The DMSO group showed the largest cell toxicity, the cells almost died in the first culture day. Many of L929 cells maintained a spherical shape and long spindle-shape. In the blank group, 75Fc and 50Fc sample media, extensions were observed from most cells that were characterized as long spindle-shape, with low cytoplasm to nuclei ratios. The viability results of L929 cells for 1, 3, 7-day exposure with the scaffolds extracted media were recorded in the Fig. 6(b). At 1-day in culture, L929 cells in 100Fc, 75Fc, 50Fc, and 100Acc extracted stained with Hoechst media demonstrated significantly higher than 25Fc sample and the DMSO group, showing no difference compared with the blank group. The number of the cells continued increasing except the DMSO group. At the 7-day, the 75Fc and 50Fcextracted media maintained highest L929 cells proliferation compared with the other samples. L929 cell proliferation of 75Fc and 50Fc demonstrated a similar pattern with the proliferation rate in the blank control. These results suggested that the 75Fc and 50Fc scaffolds with highest cellular compatibility (among the samples) could promote L929 cell proliferation.

### Cell behavior on the scaffold

Before the scaffolds applied *in vivo*, the interaction between cell and scaffold should be taken into consideration first to test as biomaterials^45^. Some material properties, including composition, swelling capacity and structure, should be a focus so as to design serviceable biomimetic scaffolds, which is critical for cell growth and maintaining cell phenotype. Following the same theory, scaffolds with different component blend ratio, pore structure and swelling capacity were designed in this research. SA was selected to optimize the single component SF scaffolds due to its similarity with polysaccharide present in ECMs. Thus, the scaffold properties will affect cellular behavior and cell fate *in vitro*.

**Figure 7:**
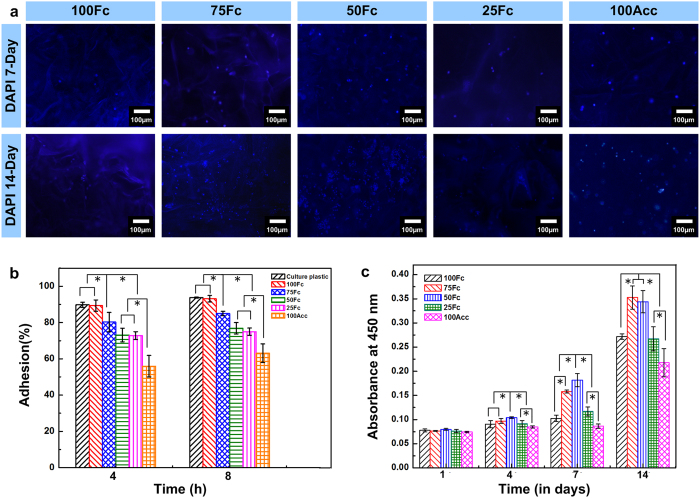
Adhesion, morphology and proliferation of L929 cells on the scaffolds. (**a**) The cells growing on the scaffolds after 7 and 14-day culture were stained with DAPI for nuclei (blue). The fluorescence of DAPI was more intense than the background fluorescence from scaffolds, thus providing the sufficient contrast for imaging. Scale bars, 100 μm. (**b**) The adhesion of L929 cells on the surface of the culture dishes and the scaffolds (n = 3 per group). (**c**) The cell viability on the scaffolds after 7 and 14-day culture (n = 3 per group). * denotes statistically significant differences. (n = 6 per group, p < 0.05).

At first, the L929 cells adhesion on the surfaces of the series of scaffolds were examined after 4- and 8- hour culture. Regardless of the fractions of the SA within the SF/SA scaffolds, all the scaffolds supported the L929 cells adhesion (Fig. 7(b)). The adhesion of L929 cells to the surfaces of the SF scaffolds was similar to that on the blank dishes. With the increasing of SA content, the adhesion of L929 cells decreased slightly, indicating that the involvement of SF in the scaffolds made the surfaces more adhesive.

And then the cellular interaction of all scaffolds was investigated according to the cell proliferation within the scaffolds. The cell number and behavior within the scaffolds was monitored by fluorescence microscope (Fig. 7(a)). DAPI staining was used to distinguish the L929 cells (blue nuclei) from the scaffolds in the dark. All scaffolds exhibited porous structure with cells attached well at 1 week, and the cells had a noted tendency to attach and proliferate along the porous structure during the culture days. Especially, the 75Fc and 50Fc scaffolds exhibited much better cell affinity, leading to the formation of many cell colonies. The inferior transparent SF scaffolds resulted in less fluorescent cells were captured in the picture. However, the proliferation of L929 cell certified these results, indicating the 75Fc and 50Fc scaffolds were much more suitable for cells attaching and proliferating.

Figure 7(c) presented the proliferation of L929 cells on 100Fc, 75Fc, 50Fc, 25Fc and 100Acc samples after 1, 4, 7 and 14 days of culture. After 1-day culture, almost the same cell proliferation was observed in the all scaffolds, which indicated that there is no difference in cell number at the initial time. The cell proliferation showed an increased tendency in the scaffolds during the cultivation process. Cell quantity on the 50Fc sample was significantly higher than that on the 100Acc samples in different days. CCK-8 results of 7- and 14- day culture indicated that all the blend samples except 25Fc exhibited much more viability compared to 100Fc and 100Acc respectively. The data proved that the blend scaffolds had better biocompatibility than the pure ones. This result was consistent with the previous research demonstrating that the protein and polysaccharides blend scaffolds could promote cell spreading and proliferation^41^. This result also suggested that the SF/SA blends scaffolds with higher swelling capacity and more regular structure provided a comfortable environment for cell proliferation and survival.

**Figure 8:**
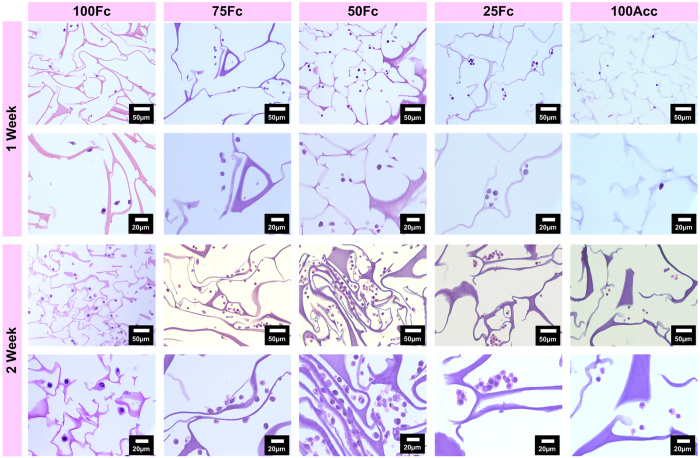
H&E staining shows the distribution of cells cultured on scaffolds after 1- and 2- week culture *in vitro*.

Figure 8 showed H&E staining of scaffolds within L929 cells after 7- and 14- day culture. Different pore structure was observed in the moist environment, and more cells were visualized within 50Fc scaffolds. With the increase of incubation time, some scaffolds showed obvious dissociation, such as 100Acc, 25Fc and 100Fc, resulting in impeding proliferation of cells. The shapes of cells in the magnifying microphotos were similar, however, the 50Fc showed high accessibility to cells due to the biomimetically high porosity and regular structure similar to skin tissue. The remarkably enhanced water stability of the crosslinked scaffolds rendered preservation of the 3D structures during the time period of cell culture. It also could be inferred that, during the cell culture time period, the different degradation behavior among the sample also bring different cell growth action. These results were consistent with the results shown in Fig. 7. Therefore, the favorable cell growth morphology and proliferation observed on the blend scaffolds mentioned above suggested the possible use for tissue engineering. The 50Fc scaffold with more prominent cell growth and proliferation was a promising biomaterial for soft tissue engineering.

## Conclusions

Stable and cytocompatible SF/SA scaffolds combining their salient features were prepared successfully by freeze dried of aqueous blended solutions. EDC crosslinked blend scaffolds 50Fc exhibited long term preservation of weight during degradation. The appropriate scaffolds were easily tuned in terms of important parameters such as pore diameter, porosity, mechanical strength, degradation and swelling capacity as per need by changing composition, thus fulfilling specific tissue engineering requirements. Noticeably, the blend scaffolds 75Fc and 50Fc with biomimetical structure similar to skin tissue remarkably enhanced cell adhension and proliferation *in vitro*. Furthermore, the whole process of preparing scaffolds was all conducted in aqueous media and performed at room temperature, offering possibility to load bioactive drugs or growth factors into the scaffold. Good cytocompatibility of these blend scaffolds combined with other advantages give an insight to these blend scaffolds mimicked the component of ECMs are promising candidates for repairing soft tissue engineering applications.

## Materials and Methods

### Materials

*Bombyx mori* raw silk fibers were purchased from Soho Biotechnology Co. Ltd. (China). SA were purchased from Shanghai Sinopharm Chemical Reagent Co. Ltd. (China), CCK-8 was purchased from Dojindo Laboratories (Japan). Roswell Park Memorial Institute Medium -1640 (RPMI-1640, Gibco) and fetal bovine serum (FBS, Gibco) were purchased from Shanghai Pufei Bio-Technology Co. Ltd. (China). Apoptosis and Necrosis Assay Kit (Hoechst and PI) and 4′,6-diamidino-2-phenylindole (DAPI) were purchased from Beyotime Institute of Biotechnology (China). Cellulose dialysis membranes (molecular-weight cut-off of 12 kDa), absolute ethanol, calcium chloride (CaCl_2_), sodium carbonate (Na_2_CO_3_), 1-ethyl-3-(3-dimethylaminopropyl) carbodiimide (EDC), 2-morpholinoethanesulfonic acid (MES) and N-hydroxysuccinimide (NHS) were purchased from Sigma (USA). All other chemicals were analytical grade and purchased from Shanghai Sinopharm Chemical Reagent Co. Ltd. (China) and used without further purification.

### Preparation of SF aqueous solution

SF solution was prepared using a chemical degumming method before dissolution and dialysis. Raw silk fibers were treated three times in 0.205 wt% Na_2_CO_3_ solutions at 98 ± 2 °C for 30 min respectively to remove sericin. After being air-dried, the refined silks were dissolved in ternary solvent CaCl_2_:CH_3_CH_2_OH:H_2_O (mole ratio = 1:2:8) at 72 ± 2 °C for 1 h. Then the mixed solution was dialyzed in deionized water for 4 days to get fibroin solution with concentration of about 3 wt%.

### Preparation of SF/SA blend scaffolds

A 2 wt% SA stock solution was obtained by dissolving SA powder in deionized water at 60 °C for 1 h. Similarly, the SF solution was diluted to 2 wt% with deionized water. Blending was performed by mixing the SF and SA stock solutions with the same concentration (2 wt%) but different volumes in a glass beaker. The final weight ratios of SF/SA in mixed solution were 100/0, 75/25, 50/50, 25/75,/0/100 (w/w), respectively (Table 1). According to the previous reports^24^, the EDC, NHS and MES were added into the solution to account for 20%, 10% and 20% weight ratio against the total weight of SF and SA in solution, respectively. The mixed solution was stirred gently at 4 °C for 30 minutes and then poured into stainless steel dish, frozen at −40 °C for 8 h, followed by lyophilization for 48 h. The 100Acc sample also prepared for stability, swelling behavior and cytocompatibility test, which obtained by immersing the 100Ac in the 3 wt% CaCl_2_ solution for 12 h.

### Pore morphology

The freeze-dried scaffolds were cut horizontally at middle depth and the cross-sectional surface was observed by SEM using a Hitachi S-4800 (Japan) with the working voltage 3 kV. Segments of the inner surfaces were prepared by fracturing with a sharp blade. The specimens were gold coated before analysis. In order to calculate pore diameter in cross-section, the border of each pore in the top layer was defined according to the gradient method^38^. Each pore area (x_1_, x_2_, … x_n_) and the area S (mm^2^) of the porous scaffold were calculated according to the limits of each pore in bitmap and the number of picture points in the whole picture. The pore diameter di (μm) of each pore, mean pore diameter 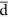
(μm), and porosity P (%) were calculated by Eqs (1)–(3):


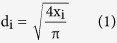



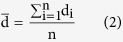



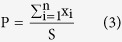


### Swelling behavior

The swelling properties were measured according to conventional gravimetric procedure. The freeze-dried SF/SA scaffolds were cut to 10 × 10 × 5 mm (length × width × height) and immersed in distilled water at 37 °C for 12 h. The wet weight of the scaffold W_t_ (g) were weighed by an electronic balance at predetermined time points after wiping excess surface liquid by filter paper. The swelling ratio SR (g/g) of the scaffolds was calculated by Eq. (4):


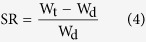


where W_t_ is the mass of the wet scaffolds at time t, and W_d_ is the mass of the dry scaffolds at time 0.

### Stability of blend scaffolds

Stability of blend scaffolds was assessed through measuring the mass loss of the scaffolds in the PBS (pH 7.4) at 37 °C. The crosslinked samples (20 × 20 × 2 mm) were incubated in 5 ml PBS for 18 days, removed and washed in distilled water three times, freeze dried and conditioned. The weights of scaffolds before and after incubation were measured on a 5 digital balance. The mass loss (ML%) was calculated according the following Eq. (5):


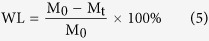


where M_t_ is the mass of the scaffolds at time t, and M_0_ is the mass of the scaffolds at time 0.

### Conformational structures analysis

The samples were prepared in KBr pellets, and then the FTIR spectroscopy of the prepared scaffold was performed using a Nicolet 6700 FTIR spectrophtometer (USA). For each measurement, each spectrum was obtained in the wave number ranging from 400 to 4000 cm^−1^.

XRD was performed by Bruker D8 Advance X-Ray Diffractometer (Germany), and CuKα radiation with a wavelength of 1.5406 Å was used. The scanning speed was 2°/min. The diffraction intensity curves with 2θ from 5 to 45° were obtained.

### Cell culture and extraction method

L929 cells were incubated in RPMI-1640 Medium supplementing with 10% FBS and 1% streptomycin-penicillin in a humidified atmosphere of 95% air and 5% CO_2_ at 37 °C. In order to measure the cell viability percentage, an extraction process was done according to the ISO 10993-5 standard test method. Scaffolds (3 mm thickness) were sterilized by immersion in ethanol 75% for 30 min, then hydrated and thoroughly rinsed with PBS. The conditioned media were obtained by incubating the scaffolds in 1 ml RPMI-1640 Medium with surface area of 3.5 ± 0.5 cm^2^ in an incubator at 37 °C for 24 h. Similar amount of the culture medium was kept in the same condition to be used as a control. Before use, the conditioned media were filtered to remove degraded scaffolds. Whereas a 10% (v/v) solution of DMSO prepared in fresh culture medium was used as a toxicity positive control. Cells were seeded at a density of 1 × 10^3^ cells/well on 96-well tissue culture polystyrene plates the day before experiments and then incubated with the 100 μl conditioned media. At each defined time point (1, 3 and 7 day), cell viability was assessed by CCK-8 assay according to the manufacturer’s instructions. The optical density (OD) was measured on a Thermo LabSystems microplate reader (MK3, USA) at 450 nm and corrected by subtracting the OD from blank wells containing only unseeded scaffolds. The cells were stained with Hoechst 33342 and PI fluorescent dye at Day 4 and then were examined by a fluorescence microscope (Olympus, IX71, Japan).

### Cell adhesion and viability analysis

To investigate the cell adhesion property of composite scaffolds, samples were cut into circular discs suitably sized for 48-well tissue culture plates. The circular matrices were sterilized with 75% alcohol under ultraviolet light overnight and then rinsed extensively three times with sterile PBS and kept in RPMI-1640 Medium for 1 hour. L929 cells were seeded at the density of 1 × 10^5^/well onto the scaffolds load in 48-well plates or cell culture dishes (as controls) and incubated at 37 °C as described above. After 4 hours and 8 hours the scaffolds were taken out carefully and washed gently with PBS (pH 7.4). By subtracting the number of cells washed out by PBS, the number of cells adhering to each hydrogel was calculated. CCK-8 assay was used to assess cell viability at Day 1, 4, 7 and 14 after cells were loaded at the density of 5 × 10^4^/well onto the SF/SA scaffolds loaded in the wells of 48-well plates. Three CCK-8 assay replicates were performed for each formulation and culture period. After 7 and 14 day culture, the scaffolds with cells were submerged for 10 minutes in PBS (pH 7.4) that was then replaced with 4% paraformaldehyde. The fixed samples were stained with 4′, 6-diamidino-2-phenylindole (DAPI), and then were examined by a fluorescence microscope (Olympus, IX71, Japan).

### Hematoxylin and Eosin (H&E) Staining

After 7 and 14 day culture, the media were aspirated, and scaffolds with L929 cells were fixed by 4% paraformaldehyde for 24 h at 4 °C. Sections were embedded in paraffin and sectioned into 5 μm slices following standard histological techniques. The sections were then positioned on a glass slide and stained with H&E for histological analyses. Finally, Sections were observed by fluorescence microscope (Olympus, IX71, Japan).

### Statistical analysis

The data are expressed as the means ± SD. All experiments were repeated at least three times, for all quantitative assays, a one-way analysis of variance (ANOVA), followed by Post hoc fisher’s LSD multiple comparison analysis, was performed. *P* < 0.05 was considered statistically significant.

## Additional Information

**How to cite this article**: Wang, Y. *et al*. A Biomimetic Silk Fibroin/Sodium Alginate Composite Scaffold for Soft Tissue Engineering. *Sci. Rep.* **6**, 39477; doi: 10.1038/srep39477 (2016).

**Publisher's note:** Springer Nature remains neutral with regard to jurisdictional claims in published maps and institutional affiliations.

## Supplementary Material

Supplementary Materials

## Figures and Tables

**Table 1:** Sample’s Compositions and Codes.

SF/SA ratio (w/w)	100/0	75/25	50/50	25/75	0/100
Uncrosslinked scaffolds	100F	75F	50F	25F	100A
EDC crosslinked scaffolds	100Fc	75Fc	50Fc	25Fc	100Ac
