# Supplementary material for: A Biomimetic Silk Fibroin/Sodium Alginate Composite Scaffold for Soft Tissue Engineering
Source: Sci Rep. 2016 Dec 20;6:39477. doi: 10.1038/srep39477 (PMC5172375; doi:10.1038/srep39477)
Supplement: Supplementary Materials [file srep39477-s1.doc]

# **Supplementary Materials：**

# **A Biomimetic Silk Fibroin/Sodium Alginate Composite Scaffold for Soft Tissue Engineering**

Yiyu Wang^1,2,3^, Xinyu Wang^1,2*^, Jian Shi^4*^, Rong Zhu^1,2^, Junhua Zhang^5^, Zongrui Zhang^1,2^, Daiwei Ma^1,2^, Yuanjing Hou^1,2^, Lin Fei^1,2^, Jing Yang^6^, Mamoru Mizuno^4^

^1^State Key Laboratory of Advanced Technology for Materials Synthesis and Processing, Wuhan University of Technology, Wuhan 430070, People’s Republic of China

^2^Biomedical Materials and Engineering Research Center of Hubei Province, Wuhan University of Technology, Wuhan 430070, People’s Republic of China

^3^Hubei Key Laboratory of Quality Control of Characteristic Fruits and Vegetables, Hubei Engineering University, Xiaogan 432000, People’s Republic of China

^4^Department of Machine Intelligence and Systems Engineering, Faculty of Systems Science and Technology, Akita Prefectural University, Akita 015-0055, Japan

^5^Life Science Technology School, Hubei Engineering University, Xiaogan 432000, People’s Republic of China

^6^School of Foreign Languages, Wuhan University of Technology, Wuhan 430070, People’s Republic of China

^*^Correspondence and requests for materials should be addressed to X. W. (wangxinyu@whut.edu.cn) or J. S. (shij@akita-pu.ac.jp)


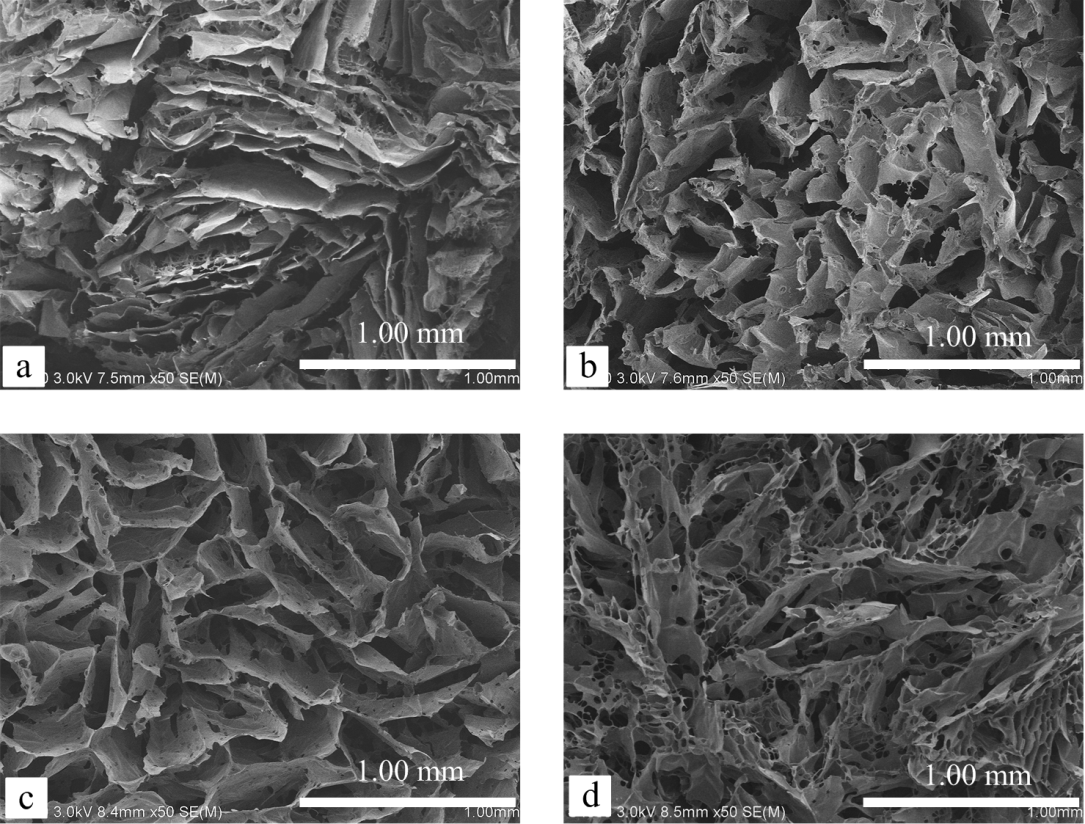


**Figure S1.** SEM images of the uncrosslinked samples with different blend ratios under freezing temperature –40 °C (a) 75F, (b) 50F, (c) 25F, (d) 100A.

Uncrosslinked SF scaffold with 2 wt. % concentration was so soft that it was difficult to obtain cross section by cutting a sample. So we supplied the SEM images of uncrosslinked scaffolds of 75F, 50F, 25F, 100A in the Fig.S1. The 75F scaffold with layered pores which were narrow and long was easy to crack, the pores of 50F scaffold turned to irregular round and became smaller, the pores of 25F scaffold was similar to 50F, while the pore size was bigger than 50F, 100A scaffold showed many small pores surrounded the bigger pores. Compared to EDC crosslinked scaffolds, the uncrosslinked scaffolds showed irregular pores with bigger pore size. These results indicated EDC reaction was helpful to obtained more regular and uniform pore structure in the scaffolds.

**
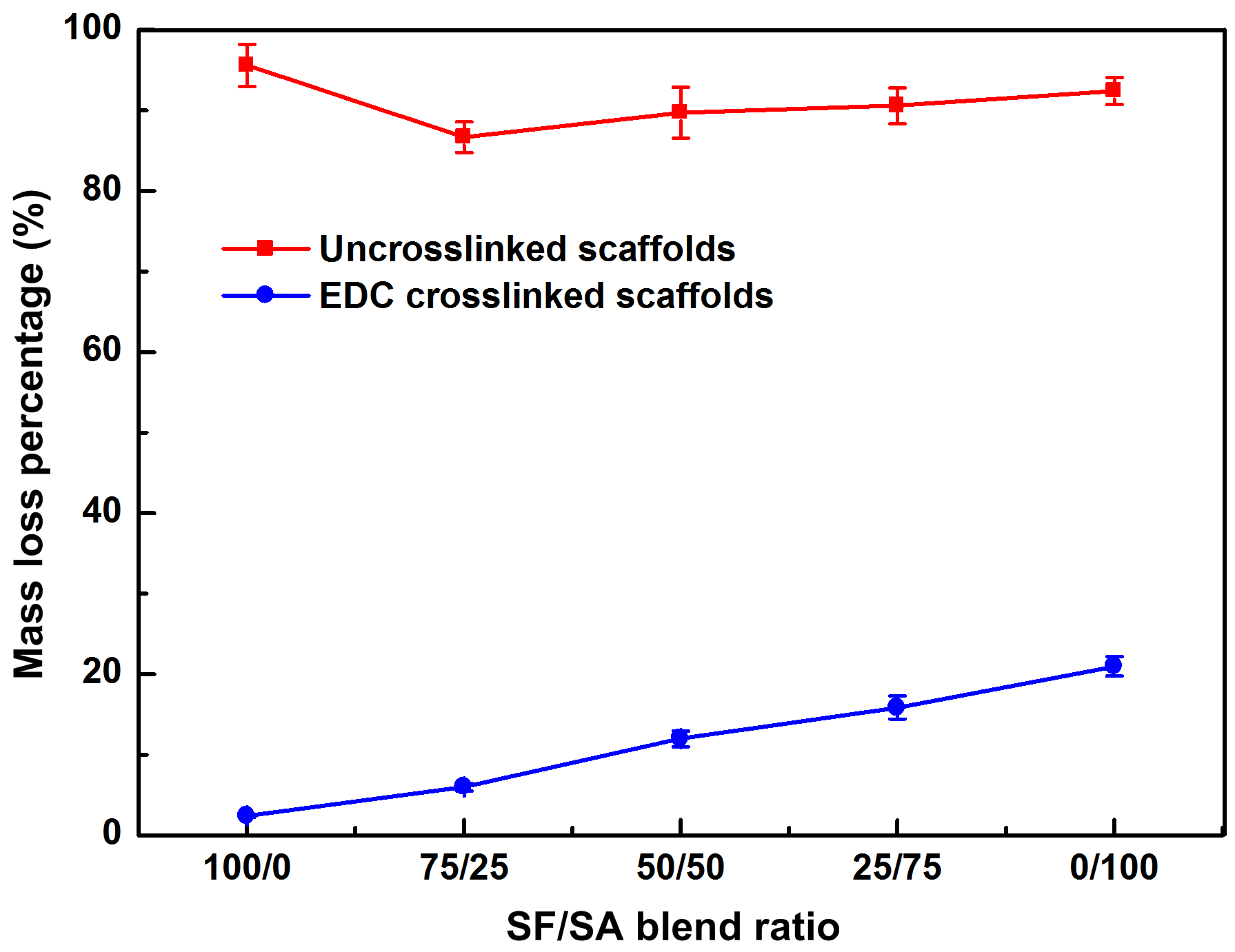
**

**Figure S2.** Mass loss of various blend ratio scaffolds after 1-day exposure with PBS.
